# Association of 42 SNPs with genetic risk for cervical cancer: an extensive meta-analysis

**DOI:** 10.1186/s12881-015-0168-z

**Published:** 2015-04-15

**Authors:** Shaoshuai Wang, Haiying Sun, Yao Jia, Fangxu Tang, Hang Zhou, Xiong Li, Jin Zhou, Kecheng Huang, Qinghua Zhang, Ting Hu, Ru Yang, Changyu Wang, Ling Xi, Dongrui Deng, Hui Wang, Shixuan Wang, Ding Ma, Shuang Li

**Affiliations:** Department of Obstetrics and Gynecology, Tongji Hospital, Tongji Medical College, Huazhong University of Science and Technology, 1095 jiefang road, Wuhan, 430030 P.R. China; Department of Gynecology & Obstetrics, the Central Hospital of Wuhan, Wuhan, 430032 P.R. China; Department of Obstetrics tumor, The tumor hospital of henan province, Zhengzhou, 450008 P.R. China

**Keywords:** Cervical cancer, Single nucleotide polymorphism, Susceptibility, Meta-analysis

## Abstract

**Background:**

A large number of single nucleotide polymorphisms (SNPs) associated with cervical cancer have been identified through candidate gene association studies and genome-wide association studies (GWAs). However, some studies have yielded different results for the same SNP. To obtain a more comprehensive understanding, we performed a meta-analysis on previously published case–control studies involving the SNPs associated with cervical cancer.

**Methods:**

Electronic searches of PubMed and Embase were conducted for all publications about the association between gene polymorphisms and cervical cancer. One-hundred and sixty-seven association studies were included in our research. For each SNP, three models (the allele, dominant and recessive effect models) were adopted in the meta-analysis. For each model, the effect summary odds ratio (OR) and 95% CI were calculated. Heterogeneity between studies was evaluated by Cochran’s Q test. If the p value of Q test was less than 0.01, a random effect model was used; otherwise, a fixed effect model was used.

**Results:**

The results of our meta-analysis showed that: (1) There were 8, 2 and 8 SNPs that were significantly associated with cervical cancer (P < 0.01) in the allele, dominant and recessive effect models, respectively. (2) rs1048943 (CYP1A1 A4889G) showed the strongest association with cervical cancer in the allele effect model (1.83[1.57, 2.13]); in addition, rs1048943 (CYP1A1 A4889G) had a very strong association in the dominant and recessive effect model. (3) 15, 11 and 10 SNPs had high heterogeneity (P < 0.01) in the three models, respectively. (4) There was no published bias for most of the SNPs according to Egger’s test (P < 0.01) and Funnel plot analysis. For some SNPs, their association with cervical cancer was only tested in a few studies and, therefore, might have been subjected to published bias. More studies on these loci are required.

**Conclusion:**

Our meta-analysis provides a comprehensive evaluation of cervical cancer association studies.

**Electronic supplementary material:**

The online version of this article (doi:10.1186/s12881-015-0168-z) contains supplementary material, which is available to authorized users.

## Background

Cervical cancer is a serious disease which affects women’s health. It is the third most common malignancy in women worldwide [1,2]. However, in China, it is the second disease only to breast cancer in the morbidity of malignancy in women. More than 200,000 women die from cervical cancer each year. China is plagued with one of the highest rates from cervical cancer in the world, and it is six times higher than other developed countries. However, trend of incidence age of pa-tients with cervical cancer gradually gets younger [3,4] Cervical cancer is a complex disease that results from the interaction between gene mutations and the environment. Epidemiological and laboratory-based studies have identified that human papilloma virus (HPV) infection contributes to cervical cancer. More than 90% cases of cervical cancer are caused by HPV infection, and type 16 and 18 are the most common types [5,6]. Although most sexually active women have been infected with HPV, nearly 90% of women with HPV infection are able to clear the virus. So only a very small proportion of women with persistent HPV infection ultimately develop into cervical cancer and it indicated that HPV infection is a necessary but not sufficient risk factor for the origin and development of cervical cancer. Consequently, host genetic differences in the effective host immune response may influence the risk for cervical cancer among those infected with HPV. Therefore, it is very important to identify the gene loci related to cervical cancer origin and progression. Over the past few decades, the genetic susceptibility of cervical cancer has been examined by candidate gene association and genome-wide association studies, and researchers have found that the most important SNP was located in 6q12, within the human leukocyte antigen (HLA), or MHC, genes [7,8]. The HLA-II (DRB1) gene contains many mutations, and these mutations result in changes of the amino acid sequence of HLA-II. Many studies have reported that HLA-II (DRB1) is strongly associated with cervical cancer. However, the structure of the DRB1 gene is complex, and thus, it is very difficult to analyze SNPs of DRB1 with the standard SNP gene effect model. At the same time, other genetic intervals and SNPs have been reported to be related to the pathogenesis of cervical cancer and to play an important role in this process. Therefore, our meta-analysis does not include SNPs in the HLA genes, but focuses on these other reported SNPs. Although researchers have had great success in their research on the gene mutations associated with cervical cancer, many problems still remains. Some studies show conflicting results for the same SNP. For example, in studies of the relationship between TNF-α-308G > A with the pathogenesis of cervical cancer, Duarte I [9] found that this SNP is significantly associated with cervical cancer (OR = 1.8, 95% CI [1.21, 2.69]). However, Gostout BS found that TNF-α-308G > A does not increase the incidence rate of cervical cancer (OR (95% CI) =0.98 [0.64, 1.50]) [10]. These controversial results may be caused by small sample sizes, racial or ethnic differences, or clinical and genetic heterogeneity. Therefore, it is very important to assess whether the combined evidence shows an association between a SNP and cervical cancer. Meta-analysis is a very effective method by which the results of many studies with small sample sizes are combined. Through this method, the relationship of some SNPs, such as TNF-α-308G > A and TNF-α-238G > A, associated with cervical cancer has been proven. TNF-α-308G > A can increase the susceptibility of cervical cancer, while TNF-α-238G > A can significantly decrease its susceptibility [11]. However, only one or two SNPs were identified in a previously published meta-analysis on SNP loci and cervical cancer. To comprehensively and systematically assess the association between all of the available SNPs and cervical cancer susceptibility, we searched the PubMed database and Embase and performed a meta-analysis on the results of the selected studies. For each SNP, three genetic models were considered: the allele, dominant and recessive effect models. We also examined the heterogeneity between studies and the existence of published bias using Egger’s test. As far as we know, this is the most detailed meta-analysis of SNPs and cervical cancer to date.

## Methods

### Data collection

**Figure 1:**
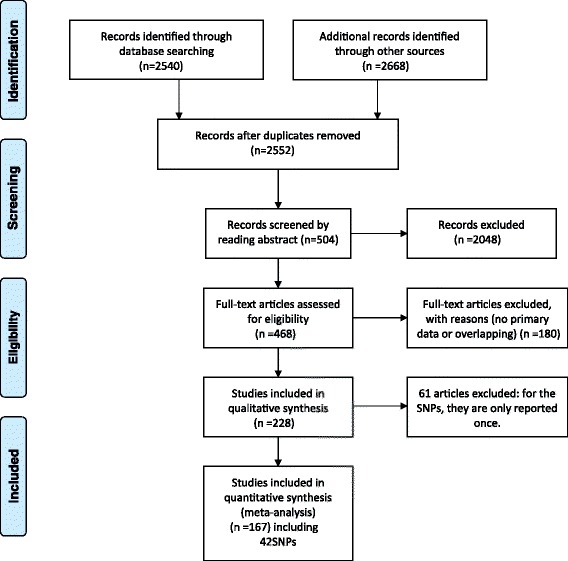
Flow chart shows study selection procedure.

The PubMed and Embase were searched for the appropriate studies using the following keywords: (polymorphism OR mutation OR single nucleotide polymorphisms OR genome-wide association study OR SNP OR GWAS) AND (cervical cancer OR cervical carcinoma). The studies to be included in the meta-analysis were selected in accordance with the following criteria: (1) the articles must have been published between January of 1990 and June of 2014; (2) the studies must employ a case–control design and must examine the association between SNPs and cervical cancer; (3) data on the SNP genotypes of patients and controls must be available; (4) the studies must be published as a full paper, not as a meeting abstract or review; and (5) NOT-HLA. For each study, we extracted the following information: the gene polymorphisms, first author, date of publication, title, population and number of cases and controls. Then, we choose those SNPs which published at least 2 times. Using these criteria, 152 papers involving 42 SNPs were selected for the meta-analysis (Figure 1).

### Selection of the genetic model

To comprehensively analyze the association between SNPs and cervical cancer, we adopted three genetic models: the allele effect model, the dominant effect model, and the recessive effect model. In these models, we assumed that each SNP marker locus has two alleles (*A* and *a*). *A* is the high-risk candidate allele, and *a* is the low-risk allele. The three models are described as follows:

1. Allele model: the effect of the *A* allele vs. the effect of the *a* allele;
2. Dominant model: If the SNP produces a cervical cancer phenotype when present in either one or two copies of the A allele, i.e., the *AA + Aa* vs. *aa* genotypes.
3. Recessive model: If only the *aa* genotype exists, the SNP produces a cervical cancer phenotype.

All meta-analysis were performed using RevMan 5.2 software. For each model, we calculated the OR value and 95% CI for the individual study. To evaluate the weight of each individual study on overall pooled OR, we performed a sensitivity analysis by sequentially removing each article at a time.

### Evaluation of heterogeneity

Cochran’s Q test was used to evaluate the heterogeneity of between- and within-study variation. In fact, Cochran’s Q test is simply a chi-square test [12]. The null hypothesis was that all studies were evaluating the same effect. Rejecting the null hypothesis meant that heterogeneity exists between studies. P < 0.01 was considered to be significant. Another indicator of heterogeneity is *I* [2], which measures the degree of inconsistency across studies. The formula is as follows: *I*^*2*^ = (Q-(k-1))/Q*100% (where *k* is the number of studies). When the value of *I*^2^ is more than 25%, 50% or 75%, low-, mid- or high-grade heterogeneity is present, respectively [13-16].

#### Evaluation of the statistical association between the identified SNPs and cervical cancer

In this meta-analysis, Cochran’s Q test was used to evaluate the heterogeneity between studies. If the Q-statistic was not significant, we considered that all of the differences between studies were caused by sampling error. Then, we selected the fixed effects model in the meta-analysis. In contrast, if the p value was significant (P < 0.01), meaning that heterogeneity exists between studies, we chose the random effects model.

#### Evaluation of publication bias

Funnel plots were used to intuitively assess publication bias. The horizontal ordinate of the Funnel plots corresponded to the study effects. If the variable was continuous, the effects are just shown as the original value; otherwise, the effects are shown as a log value. The vertical ordinate corresponds to the sample size, standard error or accuracy. The smaller the sample, the more scattered the distribution; and the larger the sample size, the more concentrated the distribution. If there is no bias, the Funnel plot is symmetrical. In contrast, if the diagram is asymmetrical, it means that publication bias exists. In addition, Egger’s test was used to quantitatively assess the symmetry of the Funnel plots [17,18]. Egger’s test cannot be used in a meta-analysis when the number of studies is less than 2. Therefore, we only used Egger’s test for SNPs with larger than or equal to 2 studies. Egger’s test was carried out using Stata 12.0 software.

## Results

In our search for eligible studies and loci, we input the aforementioned keywords into the PubMed and Embase and then obtained 2552 studies. Screened by the criteria mentioned in the data collection, 152 of these 2552 studies involving 42 SNPs were included in our meta-analysis (Additional file 1: Table S1 and Additional file 2). The Cohen’s Kappa value was 0.79(P < 0.05). Each of the 42 SNPs was reported in at least two studies. The number of studies for each locus was also counted. Fourteen SNPs were reported more than five times, and five SNPs were reported more than 10 times. The five SNPs genotypes in the cases and controls were extracted for subsequent analysis.

### Meta-analysis results for the allele effect model

For each SNP, the OR and 95% CI of the A allele (*A vs. a*) were calculated for each study, and the heterogeneity between studies was tested. In our analysis of heterogeneity, we identified 15 SNPs with a Q test P value of <0.01. Thus, for the meta-analysis of these SNPs, we used the random effects model. For the remaining SNPs that did not show heterogeneity, we used the fixed effects model. The meta-analysis showed that 8 SNPs were significantly associated with cervical cancer (P < 0.01, Table 1). Among these 8 SNPs, only 2 SNPs had heterogeneity (p < 0.00001 and p < 0.00001 for rs2279744 and rs4646903, respectively). The OR values of rs2279744 and rs4646903 were 0.64 (95% CI [0.50, 0.82]) and 1.59 (95% CI [1.39, 1.81]) in the random effects model. For the remaining 6 SNPs, the fixed effects model was used. The most significant locus was rs1048943 (CYP1A1 risk allele G/A) (p < 0.00001). The pooled summary OR based on the fixed effects model was 1.75 (95% CI [1.49, 2.05]) (Forest plot along with funnel plot was shown in Additional file 3), suggesting that the rs1048943 G allele confers susceptibility to cervical cancer. In addition, the publication bias was tested using Funnel plots and Egger’s test, and no publication bias was observed.

**Table 1:** **Meta-analysis results under the allele model**

**SNP**	**Comparison**	**Gene symbol**	**No.**	**Q**	**Q-P**	**I** ^**2**^	**Model**	**95% CI**	**P**
rs1048943	G/A	CYP1A1 A4889G	8	12.72	0.08	0.45	Fixed	1.75[1.49,2.05]	<0.00001
rs3212227	A/C	IL-12B	2	3.97	0.05	0.75	Fixed	0.75[0.64,0.87]	0.0001
rs2279744	T/G	MDM2	2	16.6	<0.00001	0.94	Random	0.64[0.50,0.82]	0.0004
rs16944	C/T	IL-1β	4	10.06	0.02	0.7	Fixed	0.79[0.69,0.90]	0.0006
rs187084	T/C	TLR9 T1486C	3	2.18	0.34	0.08	Fixed	0.84[0.75,0.95]	0.005
rs4646903	C/T	CYPA1 T6235C	8	33.46	<0.00001	0.79	Random	1.59[1.39,1.81]	0.005
rs568408	G/A	IL-12A	2	0.05	0.83	0	Fixed	0.78[0.66,0.93]	0.006
rs1800872	C/A	IL-10 C592A	5	4.69	0.32	0.15	Fixed	0.85[0.76.0.96]	0.007
rs1800795	G/C	IL-6	2	0	0.94	0	Fixed	0.69[0.51,0.92]	0.01
rs11134527	A/G	pri-miR-218	2	0.72	0.39	0	Fixed	1.11[1.02,1.21]	0.02
rs1800629	G/A	TNF-α-308	15	56.89	<0.00001	0.77	Random	0.82[0.68,0.98]	0.03
rs1801275	A/G	IL-4R	2	0.24	0.63	0	Fixed	1.18[0.98,1.42]	0.09
rs1052134	Ser326Cys	OGG1	2	0	0.96	0	Fixed	1.26[0.94,1.68]	0.12
rs603965	G/A	CCND1	6	6.6	0.25	0.24	Fixed	0.92[0.82,1.03]	0.14
rs361525	G/A	TNF-α-238	8	805.51	<0.00001	0.99	Random	2.90[0.65,12.9]	0.16
rs5742909	T/C	CTLA-318	5	18.55	0.001	0.78	Random	1.39[0.87,2.21]	0.17
rs62559044	A/T	IFN-r A874T	4	26.88	<0.00001	0.89	Random	1.10[0.94,1.29]	0.21
rs1801133	T/C	MTHFR C677T	8	35.6	<0.00001	0.8	Random	0.87[0.67,1.12]	0.27
rs352140	G/A	TLR9 G2848A	3	0.26	0.61	0	Fixed	1.09[0.92,1.29]	0.30
rs833061	T/C	VEGF T460C	2	0.39	0.53	0	Fixed	1.14[0.86,1.50]	0.35
rs11549465	C/T	HIF1A C1772T	2	11.76	0.0006	0.91	Random	0.56[0.13,2.32]	0.42
rs1805087	A/G	MTR A2756G	2	32.02	<0.00001	0.97	Random	1.95[0.34,11.18]	0.45
rs763110	T/C	FASL T844C	2	13.02	0.0003	0.92	Random	0.81[0.47,1.40]	0.45
rs1800896	G/A	IL-10 G1082A	8	5.77	0.441	0	Fixed	1.00[0.91,1.09]	0.45
rs3021097	C/T	IL-10 C819T	2	3.09	0.08	0.68	Fixed	0.92[0.72,1.16]	0.47
rs1800682	G/A	Fas	9	22.37	0.004	0.64	Random	1.06[0.90,1.24]	0.48
rs1801131	A/C	MTHFR A1298C	2	2.84	0.09	0.65	Fixed	1.08[0.86,1.36]	0.50
rs3116496	C/T	CD28	3	13.94	0.0009	0.86	Random	1.14[0.76,1.71]	0.52
rs1799864	A/G	CCR2	3	84.35	<0.00001	0.98	Random	1.30[0.46,3.69]	0.62
rs5275	C/T	COX-2	2	1.1	0.29	0.09	Fixed	0.95[0.75,1.22]	0.70
rs861539	C/T	XRCC3Codon241	5	16	0.003	0.75	Random	0.92[0.58,1.44]	0.70
rs3025039	C/T	VEGF C936T	2	1.19	0.28	0.16	Fixed	1.06[0.77,1.45]	0.72
rs2031920	c1/c2	CYP2E1	2	0.2	0.66	0	Fixed	1.03[0.71,1.50]	0.87
rsl801270	C/A	p21 codon 31	7	55.68	<0.00001	0.89	Random	0.97[0.69,1.38]	0.87
rs4404252	T/C	ICOS	2	0.08	0.77	0	Fixed	1.01[0.86,1.18]	0.93

No, number of studies; OR, combined odds ratio; CI, confidence interval; P, P value.

### Meta-analysis results for the dominant effect model

Based on the dominant model (*AA + Aa vs. aa* genotype), we tested the heterogeneity between studies. Heterogeneity was found for eleven SNPs (P < 0.01). For these SNPs, the random effects model was used in the meta-analysis. For the others that did not show heterogeneity, the fixed effects model was used. Table 2 lists all of the SNPs with dominant genetic model, and we found a significant association between two of these SNPs and cervical cancer. These two SNPs had no heterogeneity, and the fixed effects model was adopted. rs1048943 (CYP1A1) also showed the strongest association with cervical cancer in the dominant effect model (OR = 0.40, 95% CI [0.25, 0.66]). For some SNPs, although heterogeneity was observed under the allele model and the random effects model was used, they did not show heterogeneity under the dominant model; thus, the fixed effects model was then used. Some SNPs that showed a significant association with cervical cancer in the allele model did not show a significant association in the dominant model. For example, rs3212227 (IL-12B) showed a significant association with cervical cancer morbidity in the allele effect model; however, in the dominant effect model, it did not show a significant relationship with cervical cancer (OR = 0.77, 95% CI [0.56, 1.06], p = 0.11). Publication bias was tested using Funnel plots and Egger’s test. We found that CCND1 (rs603965), CD28 (rs3116496) had publication bias. This bias may have resulted because these SNPs were analyzed in only few studies or because of differences in the selection of the cases and controls.

**Table 2:** **Meta-analysis results under the dominant genetic model**

**SNP**	**Comparison**	**Gene symbol**	**No.**	**Q**	**Q-P**	**I** ^**2**^	**Model**	**95% CI**	**P**
rs1048943	GG + GA/AA	CYP1A1 A4889G	8	8.8	0.12	0.43	Fixed	0.40[0.25,0.66]	0.0002
rs11134527	AA + AG/GG	pri-miR-218	2	0	0.98	0	Fixed	1.25[1.06,1.47]	0.007
rs2279744	TT + TG/GG	MDM2	2	3.32	0.07	0.7	Fixed	0.55[0.34,0.88]	0.01
rs4646903	CC + CT/TT	CYPA1 T6235C	8	33.12	<0.0001	0.79	Random	1.65[1.12,2.43]	0.01
rs1801275	AA + AG/GG	IL-4R	2	0.15	0.69	0	Fixed	1.70[1.07,2.68]	0.02
rs1800872	CC + AC/AA	IL-10 C592A	5	1.88	0.76	0	Fixed	0.79[0.63,0.98]	0.03
rs62559044	AA + AT/TT	IFN-r A874T	4	2.47	0.48	0	Fixed	1.58[1.15,2.15]	0.04
rs1800896	GG + GA/AA	IL-10 G1082A	8	9.96	0.13	0.4	Fixed	1.19[0.99,1.44]	0.06
rs1800795	GG + GC/CC	IL-6	3	4.46	0.11	0.55	Fixed	0.52[0.26,1.07]	0.08
rs1052134	Ser326Cys	OGG1	2	1.42	0.23	0.29	Fixed	1.59[0.93,2.72]	0.09
rs352140	GG + GA/AA	TLR9 G2848A	3	4.81	0.09	0.58	Fixed	1.25[0.95,1.63]	0.10
rs3212227	AA + AC/CC	IL-12B	2	2.03	0.15	0.51	Fixed	0.77[0.56,1.06]	0.11
rs833061	TT + TC/CC	VEGF T460C	2	1.33	0.25	0.25	Fixed	1.75[0.85,3.59]	0.13
rs1805087	AA + AG/GG	MTR A2756G	2	2.72	0.1	0.63	Fixed	1.64[0.83,3.27]	0.16
rs1801133	TT + TC/CC	MTHFRC677T	8	28.67	0.0002	0.76	Random	0.81[0.60,1.10]	0.17
rs5742909	TT + TC/CC	CTLA-318	5	19.13	0.0007	0.79	Random	1.43[0.86,2.39]	0.17
rs1800629	GG + GA/AA	TNF-α-308	15	52.19	<0.00001	0.75	Random	0.82[0.57,1.18]	0.28
rs187084	TT + TC/CC	TLR9T1486C	3	2.4	0.3	0.17	Fixed	0.88[0.71,1.11]	0.29
rs16944	CC + CT/TT	IL-1β	4	16.3	0.001	0.82	Random	0.76[0.45,1.28]	0.30
rs568408	GG + GA/AA	IL-12A	2	0.64	0.42	0	Fixed	0.79[0.46,1.35]	0.39
rs1800682	GG + GA/AA	Fas	9	63.31	<0.00001	0.87	Random	1.20[0.77,1.87]	0.41
rs763110	TT + TC/CC	FASL T844C	2	13.18	0.0003	0.92	Random	0.77[0.38,1.52]	0.45
rsl801270	CC + CA/AA	p21 codon 31	7	31.99	<0.0001	0.81	Random	0.83[0.51,1.35]	0.46
rs1799864	AA + GA/GG	CCR2	3	128	<0.0001	0.98	Random	1.65[0.35,7.82]	0.52
rs361525	GG + GA/AA	TNF-α-238	8	399	<0.0001	0.98	Random	1.98[0.24,16.2]	0.53
rs3116496	CC + CT/TT	CD28	3	17.6	0.0002	0.89	Random	1.14[0.69,1.88]	0.61
rs3021097	CC + CT/TT	IL-10 C819T	2	1.27	0.26	0.21	Fixed	1.05[0.73,1.49]	0.80
rs603965	GG + GA/AA	CCND1	6	4.24	0.52	0	Fixed	0.98[0.82,1.17]	0.80
rs1801131	AA + CA/CC	MTHFR A1298C	2	2.95	0.09	0.66	Fixed	1.07[0.63,1.81]	0.81
rs3025039	CC + CT/TT	VEGF C936T	2	2.28	0.13	0.56	Fixed	1.08[0.57,2.04]	0.82
rs5275	CC + CT/TT	COX-2	2	0.03	0.86	0	Fixed	0.98[0.73,1.32]	0.91
rs4404252	TT + TC/CC	ICOS T/C	2	0.02	0.88	0	Fixed	0.99[0.64,1.54]	0.98

No, number of studies; OR, combined odds ratio; CI, confidence interval; P, P value.

### Meta-analysis results for the recessive effect model

Based on the recessive model (AA vs. Aa + aa), there were ten SNPs that showed heterogeneity, with a Q test P value of <0.01. The random effects model was used for these ten SNPs. The fixed effects model was used for the remaining SNPs. Table 3 lists the SNPs in the recessive effect model. Eight SNPs (rs1048943, rs16944, rs1048903, rs3212227, rs187084, rs352140, rs2279744 and rs568408) showed a significant association with cervical cancer. The random effects model was used for rs2279744 (OR = 0.59, 95% CI [0.42, 0.84]). SNP rs1048943, rs16944 and rs1048903 showed the most significant association with cervical cancer (0.48[0.40, 0.59], 0.58[0.45, 0.74] and 1.98[1.46, 2.69] respectively) (Forest plots along with funnel plots was shown in Additional file 3). At the same time, SNP rs1048943 also showed a relatively strong association in the allele and dominant effect model. However, the SNP rs11134527 showed a significant association in the allele model and dominant effect model but not in the recessive model, then we could infer that the rs11134527 mutation of A to G can increase the risk of cervical cancer.

**Table 3:** **Meta-analysis results under the recessive genetic model**

**SNP**	**Comparison**	**Gene symbol**	**No.**	**Q**	**Q-P**	**I** ^**2**^	**Model**	**95% CI**	**P**
rs1048943	GG/GA + AA	CYP1A1A4889G	8	14.4	0.04	0.51	Fixed	0.48[0.40,0.59]	<0.00001
rs16944	CC/CT + TT	IL-1β	4	3.37	0.34	0.11	Fixed	0.58[0.45,0.74]	<0.00001
rs4646903	TT/TC + CC	CYPA1T6235C	8	12.74	0.08	0.45	Fixed	1.98[1.46,2.69]	<0.00001
rs3212227	AA/AC + CC	IL-12B	2	1.36	0.24	0.26	Fixed	0.68[0.56,0.84]	0.0002
rs187084	TT/TC + CC	TLR9 T1486C	3	0.93	0.63	0	Fixed	0.76[0.64,0.90]	0.001
rs352140	GG/GA + AA	TLR9 G2848A	3	0.93	0.63	0	Fixed	0.76[0.64,0.90]	0.001
rs2279744	TT/TG + GG	MDM2	2	15.8	<0.00001	0.94	Random	0.59[0.42,0.84]	0.003
rs568408	GG/GA + AA	IL12A	2	0.3	0.59	0	Fixed	0.74[0.61,0.91]	0.004
rs1800872	CC/AC + AA	IL-10 C592A	5	6.67	0.15	0.4	Fixed	0.84[0.72,0.99]	0.04
rs1800896	GG/GA + AA	IL-10 G1082A	8	22.99	0.0008	0.74	Random	0.66[0.44,0.99]	0.04
rs1800795	GG/GC + CC	IL-6	2	1.35	0.24	0.26	Fixed	0.70[0.49,1.00]	0.05
rs1800629	GG/GA + AA	TNF-α-308	15	38.57	0.0004	0.64	Random	0.85[0.71,1.02]	0.08
rs361525	GG/GA + AA	TNF-α-238	8	372	<0.0001	0.98	Random	2.94[0.82,10.5]	0.10
rs3021097	CC/CT + TT	IL-10 C819T	2	1.2	0.27	0.17	Fixed	0.74[0.50,1.10]	0.14
rs11134527	AA/AG + GG	pri-miR-218	2	1.41	0.23	0.29	Fixed	1.09[0.96,1.23]	0.18
rs3116496	CC/CT + TT	CD28	3	0.18	0.92	0	Fixed	1.33[0.83,2.12]	0.24
rs1801275	AA/AG + GG	IL-4R	2	0.26	0.61	0	Fixed	1.13[0.89,1.42]	0.31
rs763110	TT/TC + CC	FASL T844C	2	3.79	0.05	0.74	Fixed	0.83[0.58,1.19]	0.31
rs1805087	AA/AG + GG	MTR A2756G	3	34.25	<0.00001	0.94	Random	1.79[0.57,5.62]	0.32
rs1801131	AA/AC + CC	MTHFRA1298C	2	0.25	0.62	0	Fixed	0.87[0.66,1.15]	0.33
rs1052134	Ser326Cys	OGG1	2	0.58	0.45	0	Fixed	1.20[0.78,1.84]	0.40
rs5275	CC/CT + TT	COX-2	2	6.18	0.01	0.84	Fixed	0.73[0.36,1.50]	0.40
rs5742909	TT/TC + CC	CTLA-318	5	2.33	0.68	0	Fixed	1.42[0.63,3.20]	0.40
rs11549465	CC/CT + TT	HIF1A C1772T	2	8	0.005	0.87	Random	0.57[0.13,2.45]	0.45
rs1799864	GG/GA + AA	CCR2	3	6.93	0.03	0.71	Fixed	0.87[0.48,1.58]	0.65
rs1800682	GG/GA + AA	Fas	9	38.81	<0.0001	0.79	Random	0.92[0.67,1.26]	0.69
rs603965	GG/GA + AA	CCND1	7	17.2	0.0009	0.65	Random	0.94[0.69,1.29]	0.70
rs3025039	CC/CT + TT	VEGF C936T	2	0	1	0	Fixed	1.08[0.72,1.62]	0.71
rs833061	TT/TC + CC	VEGF T460C	2	1.94	0.16	0.49	Fixed	1.07[0.75,1.51]	0.71
rsl801270	CC/AC + AA	p21codon 31	8	46.83	<0.00001	0.85	Random	1.07[0.71,1.62]	0.74
rs1801133	TT/TC + CC	MTHFR C677T	9	10.53	0.23	0.24	Fixed	1.03[0.85,1.25]	0.76
rs62559044	AA/AT + TT	IFN-r A874T	4	37.06	<0.00001	0.92	Random	0.92[0.41,2.09]	0.85
rs4404252	TT/TC + CC	ICOS T/C	2	0.13	0.72	0	Fixed	1.01[0.84,1.22]	0.91

No, number of studies; OR, combined odds ratio; CI, confidence interval; P, P value.

### Meta-analysis of special phenotype

During the data collection process, we noticed that some publications provided additional testing, such as genotyping for GSTM1 (positive or negative) and CYP2E1 (c1 or c2). These SNPs also were included in the meta-analysis. The results are shown in Table 4. SNPs with heterogeneity were tested using the random effects model. The fixed effects model was used for the remaining SNPs. As shown in Table 4, No SNP were significantly associated with cervical cancer (P < 0.01).

**Table 4:** **Meta-analysis results of special phenotypes**

**Gene symbol**	**Comparison**	**No.**	**Q**	**Q-P**	**I** ^**2**^	**Model**	**95% CI**	**P**
GSTM1	postive/null	16	60.49	<0.00001	0.75	Random	0.70[0.53,0.92]	0.01
GSTT1	postive/null	15	69.88	<0.00001	0.8	Random	0.69[0.60,0.97]	0.03
G4C14-to-A4T14 P73	GG/GA + AA	2	0.61	0.44	0	Fixed	0.74[0.55,1.00]	0.05
P53 codon 72	C/G	44	268	<0.00001	0.84	Random	1.14[0.99,1.34]	0.07
P53 codon 72	CC/CG + GG	44	242.19	<0.00001	0.83	Random	1.18[0.96,1.42]	0.11
XRCC3 Codon 241	CC/CT + TT	5	6.35	0.17	0.37	Fixed	0.82[0.63,1.06]	0.14
G4C14-to-A4T14 P73	G/A	2	0.1	0.76	0	Fixed	0.83[0.65,1.07]	0.16
GSTP1	AA/AG + GG	2	0.97	0.61	0	Fixed	0.88[0.69,1.11]	0.28
XRCC1 Codon 194	CC/CT + TT	9	17.78	0.02	0.55	Fixed	0.92[0.79,1.08]	0.32
XRCC1 Codon399	GG/GA + AA	12	61.12	<0.0001	0.82	Random	1.18[0.84,1.64]	0.33
XRCC1 Codon399	G/A	12	203.12	<0.00001	0.95	Random	1.23[0.80,1.91]	0.34
XRCC1 Codon194	CC + CT/TT	9	21.11	0.007	0.62	Random	0.78[0.41,1.46]	0.44
GSTP1	A/G	2	0.03	0.86	0	Fixed	0.93[0.76,1.14]	0.48
G4C14-to-A4T14 P73	GG + GA/AA	2	0.89	0.34	0	Fixed	1.23[0.60,2.52]	0.56
P53 codon 72	CC + CG/GG	44	99.69	<0.0001	0.59	Random	1.06[0.90,1.23]	0.57
XRCC1 Codon399	GG + GA/AA	11	171.77	<0.0001	0.94	Random	1.22[0.49,3.01]	0.67
GSTP1	AA + AG/GG	2	0.1	0.76	0	Fixed	0.93[0.51,1.69]	0.80
XRCC3 Codon241	CC + CT/TT	5	13.95	0.003	0.78	Random	0.88[0.27,2.85]	0.83
XRCC1 Codon194	C/T	9	25.69	<0.00001	0.69	Random	0.99[0.73,1.33]	0.93

No, number of studies; OR, combined odds ratio; CI, confidence interval; P, P value.

We performed a sensitivity analysis by sequentially removing each article at a time for the SNPs which number of studies was larger than or equal to 4 for the three models. Then we found that only CTLA-318 rs5742909, XRCC1 codon 194 in dominant genetic model and IFN-r rs62559044 4 in allele model can affect the overall pooled OR. The data can be seen in Additional file 4.

### Meta-analysis results for SNP subgroups

In our meta-analysis, some SNPs showed heterogeneity and then were subjected to subgroup analysis to explain the causes of their heterogeneity. Most SNPs were reported by only a few individual studies and were not suitable for classification into subgroups; thus, we only selected 5 SNPs for the subgroup analysis. These SNPs were P53 codon 72 Arg/Pro (44 studies), TNF-α-308 (G/A) (11 studies), GSTM1 (positive/null) (15 studies), GSTT1 (positive/null) (15 studies) and XRCC1 Condon399 (G/A) (12 studies). The results are listed in Table 5. For P53 codon 72 Arg/Pro, the 44 studies were divided into two subgroups: the Asian group (17 studies) and the Caucasians group (11 studies). We selected the random effects model if the SNP had heterogeneity; otherwise, we selected the fixed effects model. We found that this SNP was significant associated with cervical cancer in the allele effect model and that the Arg allele increased the susceptibility of cervical cancer in the Caucasians groups but did not show a significant association in the Asian group. The remaining 4 SNPs did not show significantly association with cervical cancer in the two group in the three effect model (All p values were larger than 0.01). In addition, we found that some SNPs had heterogeneity when considering the total population but did not have heterogeneity when divided into subgroups. This phenomenon indicates that population size is one reason for heterogeneity. However, if the SNPs also showed heterogeneity in the subgroup, other reasons, such as race, may account for the presence of heterogeneity.

**Table 5:** **Meta-analysis results of subgroups**

**Gene symbol**	**Subgroup**	**Comparison**	**No.**	**Q-P**	**Model**	**OR 95% CI**	**P**
GSTT1	Total	postive/null	15	<0.00001	Random	0.69[0.60,0.79]	0.03
	Caucasians	postive/null	3	0.99	Fixed	0.89[0.69,1.30]	0.54
	Asian	postive/null	10	<0.00001	Random	0.80[0.56,1.14]	0.22
GSTM1	Total	postive/null	16	<0.00001	Random	0.70[0.53,0.92]	0.01
	Caucasians	postive/null	6	<0.00001	Random	0.51[0.25,1.03]	0.06
	Asian	postive/null	10	<0.00001	Random	0.79[0.60,1.02]	0.07
P53 codon 72	Total	C/G	44	<0.00001	Random	1.14[0.99,1.34]	0.07
	Caucasians	C/G	11	0.02	Fixed	1.14[1.02,1.27]	0.02
	Asian	C/G	17	<0.00001	Random	1.20[0.92,1.58]	0.18
	Total	CC + GC/GG	44	<0.0001	Random	1.06[0.90,1.23]	0.57
	Caucasians	CC + GC/GG	11	0.49	Fixed	1.02[0.82,1.28]	0.83
	Asian	CC + GC/GG	17	0.0009	Random	0.95[0.74,1.22]	0.70
	Total	CC/GC + GG	44	<0.00001	Random	1.18[0.96,1.42]	0.11
	Caucasians	CC/GC + GG	11	0.02	Fixed	1.26[1.08,1.46]	0.003
	Asian	CC/GC + GG	17	<0.00001	Random	1.24[0.92,1.67]	0.15
TNF-α-308	Total	G/A	15	<0.00001	Random	0.82[0.68,0.98]	0.03
	Caucasians	G/A	6	<0.00001	Random	0.83[0.61,1.13]	0.25
	Asian	G/A	5	0.0003	Random	0.72[0.45,1.17]	0.18
	Total	GG + GA/AA	15	<0.00001	Random	0.82[0.57,1.18]	0.28
	Caucasians	GG + GA/AA	6	<0.00001	Random	0.74[0.39,1.39]	0.35
	Asian	GG + GA/AA	5	0.09	Fixed	0.97[0.79,1.19]	0.76
	Total	GG/GA + AA	15	0.0004	Random	0.85[0.71,1.02]	0.08
	Caucasians	GG/GA + AA	6	0.003	Random	0.87[0.68,1.11]	0.27
	Asian	GG/GA + AA	5	0.002	Random	0.76[0.46,1.27]	0.29
XRCC1 Codon 399	Total	G/A	12	<0.00001	Random	1.23[0.80,1.91]	0.34
	Caucasians	G/A	3	0.004	Random	0.98[0.60,1.59]	0.92
	Asian	G/A	7	<0.00001	Random	1.45[0.83,2.53]	0.19
	Total	GG + GA/AA	11	<0.0001	Random	1.22[0.49,3.01]	0.67
	Caucasians	GG + GA/AA	3	0.46	Fixed	0.71[0.50,1.01]	0.06
	Asian	GG + GA/AA	7	<0.0001	Random	2.01[0.76,5.31]	0.16
	Total	GG/GA + AA	12	<0.0001	Random	1.18[0.84,1.64]	0.33
	Caucasians	GG/GA + AA	4	0.001	Random	1.13[0.56,2.29]	0.74
	Asian	GG/GA + AA	7	<0.0001	Random	1.31[0.88,1.95]	0.18

No, number of studies; OR, combined odds ratio; CI, confidence interval; P, P value.

## Discussion

In recent years, many SNPs have been demonstrated to be associated with cervical cancer by candidate gene association studies and GWAS. For an individual SNP, many studies show inconsistent results, which are sometimes even contradictory, perhaps due to false positives, false negatives, or race or population differences. Meta-analysis is a powerful tool that can increase statistical power by pooling the results of independent studies. In this paper, we carried out a comprehensive and systematic meta-analysis to assess the relationship between 42 SNPs and the risk of cervical cancer. We used three genetic models: the allele model, dominant model and recessive model. Our meta-analysis results showed that 8, 2 and 8 SNPs showed significant associations with cervical cancer in each model, respectively. In the three effect model, SNP rs1048943 (CYP1A1 G/A) all demonstrated the highly significant association with cervical cancer (All p values < 0.00001). In the recessive model, the SNPs rs16944 (IL-1βC/T) and rs4646903 (CYPA1 T/C) also showed highly significant association with cervical cancer (All p values < 0.00001). The finding of positive SNPs is very important for the prevention, treatment and prognosis of cervical cancer. For example, the CYP1A1 (cytochrome P450) gene, which is present in 15q22-24, is a key metabolic enzyme that activates polycyclic aromatic hydrocarbon, catalyzes the oxidation of foreign compounds in the body, transforms inactive carcinogens into electrophilic compounds and promotes the formation of DNA adducts, thus generating immunotoxicity and, finally, cancer. It has been discovered that the polymorphism of CYP1A1 is associated with many cancers, such as lung cancer [19], esophageal cancer [20], endometrial cancer [21], and cervical cancer [22]. In another example, the mutation of IL-12B rs3212227 disrupts immune regulation in the host, leads to persistent HPV infection and promotes the occurrence of cervical cancer [23]. IL-1β is an inflammatory molecule that promotes angiogenesis and inhibits the immune response of the host. In many tumors, the patient prognosis is poor if the expression of IL-1β is high. SNP rs16944 increases the expression of IL-1β, thus increasing the susceptibility of cancer [24,25]. For the 42 SNPs identified in our meta-analysis, publication bias was tested using Funnel plots and Egger’s test. CCND1 (dominant model, P = 0.006) and CD28 (dominant model, P = 0.083) had significant publication bias. However, these three SNPs had no significant association with cervical cancer. In other words, their publication bias had no influence on our positive results.

## Conclusion

In conclusion, our meta-analysis, which identified 42 SNPs, showed the pooling effects. Through this meta-analysis, SNP loci that are associated with cervical cancer were discovered. However, some of these SNPs were reported so few times that it was difficult to assess the significance of their association with cervical cancer. We plan to continue our search for articles on SNPs and cervical cancer and will update our database accordingly.

## Additional files

Additional file 1: Table S1. The details of 42 SNPs selected for the meta-analysis. Additional file 2:
**The 152 studies included into the** **meta-analysis.**
Additional file 3:
**Forest plots and Funnel plots.**
Additional file 4:
**Sensitivity analysis.**

## Abbreviations

SNP: Single nucleotide polymorphisms
GWAS: Genome-wide association studies
OR: Odds ratio
CI: Confidence interval
