# Supplementary material for: Association of 42 SNPs with genetic risk for cervical cancer: an extensive meta-analysis
Source: BMC Med Genet. 2015 Apr 15;16:25. doi: 10.1186/s12881-015-0168-z (PMC4436168; doi:10.1186/s12881-015-0168-z)
Supplement: Additional file 2: — The 152 studies included into the meta-analysis. [file 12881_2015_168_MOESM2_ESM.docx]

**References**

1. Djansugurova LB, Perfilyeva AV, Zhunusova GS, Djantaeva KB, Iksan OA, Khussainova EM: **The determination of genetic markers of age-related cancer pathologies in populations from Kazakhstan**. *Front Genet* 2013, **4**:70.
2. Jeon YT, Kim JW, Song JH, Park NH, Song YS, Kang SB, Lee HP: **Cyclin D1 G870A polymorphism and squamous cell carcinoma of the uterine cervix in Korean women**. *Cancer Lett* 2005, **223**(2):259-263.
3. Ni J, Wang M, Wang M, Fu S, Zhou D, Zhang Z, Han S: **CCND1 G870A polymorphism and cervical cancer risk: a case-control study and meta-analysis**. *J Cancer Res Clin Oncol* 2011, **137**(3):489-494.
4. Satinder K, Chander SR, Pushpinder K, Indu G, Veena J: **Cyclin D1 (G870A) polymorphism and risk of cervix cancer: a case control study in north Indian population**. *Mol Cell Biochem* 2008, **315**(1-2):151-157.
5. Thakur N, Hussain S, Kohaar I, Tabassum R, Nasare V, Tiwari P, Batra S, Bhambhani S, Das BC, Basir SF *et al*: **Genetic variant of CCND1: association with HPV-mediated cervical cancer in Indian population**. *Biomarkers* 2009, **14**(4):219-225.
6. Warchol T, Kruszyna L, Lianeri M, Roszak A, Jagodzinski PP: **Distribution of CCND1 A870G polymorphism in patients with advanced uterine cervical carcinoma**. *Pathol Oncol Res* 2011, **17**(1):133-137.
7. Chatterjee K, Dandara C, Hoffman M, Williamson AL: **CCR2-V64I polymorphism is associated with increased risk of cervical cancer but not with HPV infection or pre-cancerous lesions in African women**. *Bmc Cancer* 2010, **10**:278.
8. Zheng B, Wiklund F, Gharizadeh B, Sadat M, Gambelunghe G, Hallmans G, Dillner J, Wallin KL, Ghaderi M: **Genetic polymorphism of chemokine receptors CCR2 and CCR5 in Swedish cervical cancer patients**. *Anticancer Res* 2006, **26**(5B):3669-3674.
9. Ivansson EL, Gustavsson IM, Magnusson JJ, Steiner LL, Magnusson PK, Erlich HA, Gyllensten UB: **Variants of chemokine receptor 2 and interleukin 4 receptor, but not interleukin 10 or Fas ligand, increase risk of cervical cancer**. *Int J Cancer* 2007, **121**(11):2451-2457.
10. Chen X, Li H, Qiao Y, Yu D, Guo H, Tan W, Lin D: **Association of CD28 gene polymorphism with cervical cancer risk in a Chinese population**. *Int J Immunogenet* 2011, **38**(1):51-54.
11. Pawlak E, Karabon L, Wlodarska-Polinska I, Jedynak A, Jonkisz A, Tomkiewicz A, Kornafel J, Stepien M, Ignatowicz A, Lebioda A *et al*: **Influence of CTLA-4/CD28/ICOS gene polymorphisms on the susceptibility to cervical squamous cell carcinoma and stage of differentiation in the Polish population**. *Hum Immunol* 2010, **71**(2):195-200.
12. Ivansson EL, Juko-Pecirep I, Gyllensten UB: **Interaction of immunological genes on chromosome 2q33 and IFNG in susceptibility to cervical cancer**. *Gynecol Oncol* 2010, **116**(3):544-548.
13. Pandey S, Mittal RD, Srivastava M, Srivastava K, Mittal B: **Cyclooxygenase-2 gene polymorphisms and risk of cervical cancer in a North Indian population**. *Int J Gynecol Cancer* 2010, **20**(4):625-630.
14. Lee TS, Jeon YT, Kim JW, Park NH, Kang SB, Lee HP, Song YS: **Lack of association of the cyclooxygenase-2 and inducible nitric oxide synthase gene polymorphism with risk of cervical cancer in Korean population**. *Ann N Y Acad Sci* 2007, **1095**:134-142.
15. Alsbeih G, Al-Harbi N, El-Sebaie M, Al-Badawi I: **HPV prevalence and genetic predisposition to cervical cancer in Saudi Arabia**. *Infect Agent Cancer* 2013, **8**(1):15.
16. Rahimifar S, Erfani N, Sarraf Z, Ghaderi A: **ctla-4 gene variations may influence cervical cancer susceptibility**. *Gynecol Oncol* 2010, **119**(1):136-139.
17. Su TH, Chang TY, Lee YJ, Chen CK, Liu HF, Chu CC, Lin M, Wang PT, Huang WC, Chen TC *et al*: **CTLA-4 gene and susceptibility to human papillomavirus-16-associated cervical squamous cell carcinoma in Taiwanese women**. *Carcinogenesis* 2007, **28**(6):1237-1240.
18. Sugawara T, Nomura E, Sagawa T, Sakuragi N, Fujimoto S: **CYP1A1 polymorphism and risk of gynecological malignancy in Japan**. *Int J Gynecol Cancer* 2003, **13**(6):785-790.
19. Joseph T, Chacko P, Wesley R, Jayaprakash PG, James FV, Pillai MR: **Germline genetic polymorphisms of CYP1A1, GSTM1 and GSTT1 genes in Indian cervical cancer: associations with tumor progression, age and human papillomavirus infection**. *Gynecol Oncol* 2006, **101**(3):411-417.
20. Gutman G, Morad T, Peleg B, Peretz C, Bar-Am A, Safra T, Grisaru D: **CYP1A1 and CYP2D6 gene polymorphisms in Israeli Jewish women with cervical cancer**. *Int J Gynecol Cancer* 2009, **19**(8):1300-1302.
21. Taskiran C, Aktas D, Yigit-Celik N, Alikasifoglu M, Yuce K, Tuncbilek E, Ayhan A: **CYP1A1 gene polymorphism as a risk factor for cervical intraepithelial neoplasia and invasive cervical cancer**. *Gynecol Oncol* 2006, **101**(3):503-506.
22. Abbas M, Srivastava K, Imran M, Banerjee M: **Association of CYP1A1 gene variants rs4646903 (T>C) and rs1048943 (A>G) with cervical cancer in a North Indian population**. *Eur J Obstet Gynecol Reprod Biol* 2014, **176**:68-74.
23. Roszak A, Lianeri M, Sowinska A, Jagodzinski PP: **CYP1A1 Ile462Val Polymorphism as a Risk Factor in Cervical Cancer Development in the Polish Population**. *Mol Diagn Ther* 2014, **18**(4):445-450.
24. Juarez-Cedillo T, Vallejo M, Fragoso JM, Hernandez-Hernandez DM, Rodriguez-Perez JM, Sanchez-Garcia S, Del CGM, Garcia-Carranca A, Mohar-Betancourt A, Granados J *et al*: **The risk of developing cervical cancer in Mexican women is associated to CYP1A1 MspI polymorphism**. *Eur J Cancer* 2007, **43**(10):1590-1595.
25. von Keyserling H, Bergmann T, Schuetz M, Schiller U, Stanke J, Hoffmann C, Schneider A, Lehrach H, Dahl A, Kaufmann AM: **Analysis of 4 single-nucleotide polymorphisms in relation to cervical dysplasia and cancer development using a high-throughput ligation-detection reaction procedure**. *Int J Gynecol Cancer* 2011, **21**(9):1664-1671.
26. Kim JW, Lee CG, Park YG, Kim KS, Kim IK, Sohn YW, Min HK, Lee JM, Namkoong SE: **Combined analysis of germline polymorphisms of p53, GSTM1, GSTT1, CYP1A1, and CYP2E1: relation to the incidence rate of cervical carcinoma**. *Cancer* 2000, **88**(9):2082-2091.
27. Nishino K, Sekine M, Kodama S, Sudo N, Aoki Y, Seki N, Tanaka K: **Cigarette smoking and glutathione S-transferase M1 polymorphism associated with risk for uterine cervical cancer**. *J Obstet Gynaecol Res* 2008, **34**(6):994-1001.
28. Sierra-Torres CH, Au WW, Arrastia CD, Cajas-Salazar N, Robazetti SC, Payne DA, Tyring SK: **Polymorphisms for chemical metabolizing genes and risk for cervical neoplasia**. *Environ Mol Mutagen* 2003, **41**(1):69-76.
29. Chatterjee K, Engelmark M, Gyllensten U, Dandara C, van der Merwe L, Galal U, Hoffman M, Williamson AL: **Fas and FasL gene polymorphisms are not associated with cervical cancer but differ among Black and Mixed-ancestry South Africans**. *BMC Res Notes* 2009, **2**:238.
30. Kang S, Dong SM, Seo SS, Kim JW, Park SY: **FAS -1377 G/A polymorphism and the risk of lymph node metastasis in cervical cancer**. *Cancer Genet Cytogenet* 2008, **180**(1):1-5.
31. Kordi TD, Sobti RC, Shekari M: **Association of Fas-670 gene polymorphism with risk of cervical cancer in North Indian population**. *Clin Exp Obstet Gynecol* 2008, **35**(3):183-186.
32. Lai HC, Sytwu HK, Sun CA, Yu MH, Yu CP, Liu HS, Chang CC, Chu TY: **Single nucleotide polymorphism at Fas promoter is associated with cervical carcinogenesis**. *Int J Cancer* 2003, **103**(2):221-225.
33. Sun T, Zhou Y, Li H, Han X, Shi Y, Wang L, Miao X, Tan W, Zhao D, Zhang X *et al*: **FASL -844C polymorphism is associated with increased activation-induced T cell death and risk of cervical cancer**. *J Exp Med* 2005, **202**(7):967-974.
34. Ueda M, Terai Y, Kanda K, Kanemura M, Takehara M, Yamaguchi H, Nishiyama K, Yasuda M, Ueki M: **Fas gene promoter -670 polymorphism in gynecological cancer**. *Int J Gynecol Cancer* 2006, **16 Suppl 1**:179-182.
35. Zoodsma M, Nolte IM, Schipper M, Oosterom E, van der Steege G, de Vries EG, Te MG, van der Zee AG: **Interleukin-10 and Fas polymorphisms and susceptibility for (pre)neoplastic cervical disease**. *Int J Gynecol Cancer* 2005, **15 Suppl 3**:282-290.
36. Zucchi F, Da SI, Ribalta JC, de Souza NC, Speck NM, Girao MJ, Brenna SM, Syrjanen KJ: **Fas/CD95 promoter polymorphism gene and its relationship with cervical carcinoma**. *Eur J Gynaecol Oncol* 2009, **30**(2):142-144.
37. Settheetham-Ishida W, Yuenyao P, Kularbkaew C, Settheetham D, Ishida T: **Glutathione S-transferase (GSTM1 and GSTT1) polymorphisms in cervical cancer in Northeastern Thailand**. *Asian Pac J Cancer Prev* 2009, **10**(3):365-368.
38. Agodi A, Barchitta M, Cipresso R, Marzagalli R, La Rosa N, Caruso M, Castiglione MG, Travali S: **Distribution of p53, GST, and MTHFR polymorphisms and risk of cervical intraepithelial lesions in sicily**. *Int J Gynecol Cancer* 2010, **20**(1):141-146.
39. Chen C, Madeleine MM, Weiss NS, Daling JR: **Glutathione S-transferase M1 genotypes and the risk of squamous carcinoma of the cervix: a population-based case-control study**. *Am J Epidemiol* 1999, **150**(6):568-572.
40. Goodman MT, McDuffie K, Hernandez B, Bertram CC, Wilkens LR, Guo C, Seifried A, Killeen J, Le Marchand L: **CYP1A1, GSTM1, and GSTT1 polymorphisms and the risk of cervical squamous intraepithelial lesions in a multiethnic population**. *Gynecol Oncol* 2001, **81**(2):263-269.
41. Lee SA, Kim JW, Roh JW, Choi JY, Lee KM, Yoo KY, Song YS, Kang D: **Genetic polymorphisms of GSTM1, p21, p53 and HPV infection with cervical cancer in Korean women**. *Gynecol Oncol* 2004, **93**(1):14-18.
42. Niwa Y, Hirose K, Nakanishi T, Nawa A, Kuzuya K, Tajima K, Hamajima N: **Association of the NAD(P)H: quinone oxidoreductase C609T polymorphism and the risk of cervical cancer in Japanese subjects**. *Gynecol Oncol* 2005, **96**(2):423-429.
43. Palma S, Novelli F, Padua L, Venuti A, Prignano G, Mariani L, Cozzi R, Tirindelli D, Testa A: **Interaction between glutathione-S-transferase polymorphisms, smoking habit, and HPV infection in cervical cancer risk**. *J Cancer Res Clin Oncol* 2010, **136**(7):1101-1109.
44. Sharma A, Sharma JK, Murthy NS, Mitra AB: **Polymorphisms at GSTM1 and GSTT1 gene loci and susceptibility to cervical cancer in Indian population**. *Neoplasma* 2004, **51**(1):12-16.
45. Singh H, Sachan R, Devi S, Pandey SN, Mittal B: **Association of GSTM1, GSTT1, and GSTM3 gene polymorphisms and susceptibility to cervical cancer in a North Indian population**. *Am J Obstet Gynecol* 2008, **198**(3):301-303.
46. Sobti RC, Kaur S, Kaur P, Singh J, Gupta I, Jain V, Nakahara A: **Interaction of passive smoking with GST (GSTM1, GSTT1, and GSTP1) genotypes in the risk of cervical cancer in India**. *Cancer Genet Cytogenet* 2006, **166**(2):117-123.
47. Ueda M, Toji E, Nunobiki O, Sato N, Izuma S, Torii K, Okamoto Y, Noda S: **Germline polymorphisms of glutathione-S-transferase GSTM1, GSTT1 and p53 codon 72 in cervical carcinogenesis**. *Hum Cell* 2010, **23**(4):119-125.
48. Jee SH, Lee JE, Kim S, Kim JH, Um SJ, Lee SJ, Namkoong SE, Park JS: **GSTP1 polymorphism, cigarette smoking and cervical cancer risk in Korean women**. *Yonsei Med J* 2002, **43**(6):712-716.
49. de Carvalho CR, Da SI, Pereira JS, de Souza NC, Focchi GR, Ribalta JC: **Polymorphisms of p53, GSTM1 and GSTT1, and HPV in uterine cervix adenocarcinoma**. *Eur J Gynaecol Oncol* 2008, **29**(6):590-593.
50. Kim YH, Park IA, Park WY, Kim JW, Kim SC, Park NH, Song YS, Kang SB: **Hypoxia-inducible factor 1alpha polymorphisms and early-stage cervical cancer**. *Int J Gynecol Cancer* 2011, **21**(1):2-7.
51. Konac E, Onen HI, Metindir J, Alp E, Biri AA, Ekmekci A: **An investigation of relationships between hypoxia-inducible factor-1 alpha gene polymorphisms and ovarian, cervical and endometrial cancers**. *Cancer Detect Prev* 2007, **31**(2):102-109.
52. Shekari M, Kordi-Tamandani DM, MalekZadeh K, Sobti RC, Karimi S, Suri V: **Effect of anti-inflammatory (IL-4, IL-10) cytokine genes in relation to risk of cervical carcinoma**. *Am J Clin Oncol* 2012, **35**(6):514-519.
53. Roh JW, Kim MH, Seo SS, Kim SH, Kim JW, Park NH, Song YS, Park SY, Kang SB, Lee HP: **Interleukin-10 promoter polymorphisms and cervical cancer risk in Korean women**. *Cancer Lett* 2002, **184**(1):57-63.
54. Govan VA, Carrara HR, Sachs JA, Hoffman M, Stanczuk GA, Williamson AL: **Ethnic differences in allelic distribution of IFN-g in South African women but no link with cervical cancer**. *J Carcinog* 2003, **2**(1):3.
55. Fernandes AP, Goncalves MA, Simoes RT, Mendes-Junior CT, Duarte G, Donadi EA: **A pilot case-control association study of cytokine polymorphisms in Brazilian women presenting with HPV-related cervical lesions**. *Eur J Obstet Gynecol Reprod Biol* 2008, **140**(2):241-244.
56. Matsumoto K, Oki A, Satoh T, Okada S, Minaguchi T, Onuki M, Ochi H, Nakao S, Sakurai M, Abe A *et al*: **Interleukin-10 -1082 gene polymorphism and susceptibility to cervical cancer among Japanese women**. *Jpn J Clin Oncol* 2010, **40**(11):1113-1116.
57. Wang Q, Zhang C, Walayat S, Chen HW, Wang Y: **Association between cytokine gene polymorphisms and cervical cancer in a Chinese population**. *Eur J Obstet Gynecol Reprod Biol* 2011, **158**(2):330-333.
58. Barbisan G, Perez LO, Contreras A, Golijow CD: **TNF-alpha and IL-10 promoter polymorphisms, HPV infection, and cervical cancer risk**. *Tumour Biol* 2012, **33**(5):1549-1556.
59. Stanczuk GA, Sibanda EN, Perrey C, Chirara M, Pravica V, Hutchinson IV, Tswana SA: **Cancer of the uterine cervix may be significantly associated with a gene polymorphism coding for increased IL-10 production**. *Int J Cancer* 2001, **94**(6):792-794.
60. Singh H, Jain M, Sachan R, Mittal B: **Association of TNFA (-308G>A) and IL-10 (-819C>T) promoter polymorphisms with risk of cervical cancer**. *Int J Gynecol Cancer* 2009, **19**(7):1190-1194.
61. Gangwar R, Pandey S, Mittal RD: **Association of interferon-gamma +874A polymorphism with the risk of developing cervical cancer in north-Indian population**. *BJOG* 2009, **116**(12):1671-1677.
62. Kordi TM, Sobti RC, Shekari M, Mukesh M, Suri V: **Expression and polimorphism of IFN-gamma gene in patients with cervical cancer**. *Exp Oncol* 2008, **30**(3):224-229.
63. Roszak A, Mostowska A, Sowinska A, Lianeri M, Jagodzinski PP: **Contribution of IL12A and IL12B polymorphisms to the risk of cervical cancer**. *Pathol Oncol Res* 2012, **18**(4):997-1002.
64. Chen X, Han S, Wang S, Zhou X, Zhang M, Dong J, Shi X, Qian N, Wang X, Wei Q *et al*: **Interactions of IL-12A and IL-12B polymorphisms on the risk of cervical cancer in Chinese women**. *Clin Cancer Res* 2009, **15**(1):400-405.
65. Al-Tahhan MA, Etewa RL, El BM: **Association between circulating interleukin-1 beta (IL-1beta) levels and IL-1beta C-511T polymorphism with cervical cancer risk in Egyptian women**. *Mol Cell Biochem* 2011, **353**(1-2):159-165.
66. Qian N, Chen X, Han S, Qiang F, Jin G, Zhou X, Dong J, Wang X, Shen H, Hu Z: **Circulating IL-1beta levels, polymorphisms of IL-1B, and risk of cervical cancer in Chinese women**. *J Cancer Res Clin Oncol* 2010, **136**(5):709-716.
67. Singh H, Sachan R, Goel H, Mittal B: **Genetic variants of interleukin-1RN and interleukin-1beta genes and risk of cervical cancer**. *BJOG* 2008, **115**(5):633-638.
68. Kang S, Kim JW, Park NH, Song YS, Park SY, Kang SB, Lee HP: **Interleukin-1 beta-511 polymorphism and risk of cervical cancer**. *J Korean Med Sci* 2007, **22**(1):110-113.
69. Calhoun ES, McGovern RM, Janney CA, Cerhan JR, Iturria SJ, Smith DI, Gostout BS, Persing DH: **Host genetic polymorphism analysis in cervical cancer**. *Clin Chem* 2002, **48**(8):1218-1224.
70. Gangwar R, Mittal B, Mittal RD: **Association of interleukin-6 -174G>C promoter polymorphism with risk of cervical cancer**. *Int J Biol Markers* 2009, **24**(1):11-16.
71. Nogueira DSN, Brenna SM, Campos F, Syrjanen KJ, Baracat EC, Silva ID: **Interleukin-6 polymorphisms and the risk of cervical cancer**. *Int J Gynecol Cancer* 2006, **16**(3):1278-1282.
72. Meissner RV, Barbosa RN, Fernandes JV, Galvao TM, Galvao AF, Oliveira GH: **No association between SNP309 promoter polymorphism in the MDM2 and cervical cancer in a study from northeastern Brazil**. *Cancer Detect Prev* 2007, **31**(5):371-374.
73. Singhal P, Hussain S, Thakur N, Batra S, Salhan S, Bhambani S, Bharadwaj M: **Association of MDM2 and p53 polymorphisms with the advancement of cervical carcinoma**. *Dna Cell Biol* 2013, **32**(1):19-27.
74. Kang S, Kim JW, Kang GH, Park NH, Song YS, Kang SB, Lee HP: **Polymorphism in folate- and methionine-metabolizing enzyme and aberrant CpG island hypermethylation in uterine cervical cancer**. *Gynecol Oncol* 2005, **96**(1):173-180.
75. Kohaar I, Kumar J, Thakur N, Hussain S, Niyaz MK, Das BC, Sengupta S, Bharadwaj M: **Homocysteine levels are associated with cervical cancer independent of methylene tetrahydrofolate reductase gene (MTHFR) polymorphisms in Indian population**. *Biomarkers* 2010, **15**(1):61-68.
76. Lambropoulos AF, Agorastos T, Foka ZJ, Chrisafi S, Constantinidis TC, Bontis J, Kotsis A: **Methylenetetrahydrofolate reductase polymorphism C677T is not associated to the risk of cervical dysplasia**. *Cancer Lett* 2003, **191**(2):187-191.
77. Mostowska A, Myka M, Lianeri M, Roszak A, Jagodzinski PP: **Folate and choline metabolism gene variants and development of uterine cervical carcinoma**. *Clin Biochem* 2011, **44**(8-9):596-600.
78. Shekari M, Sobti RC, Kordi TD, Suri V: **Impact of methylenetetrahydrofolate reductase (MTHFR) codon (677) and methionine synthase (MS) codon (2756) on risk of cervical carcinogenesis in North Indian population**. *Arch Gynecol Obstet* 2008, **278**(6):517-524.
79. Sull JW, Jee SH, Yi S, Lee JE, Park JS, Kim S, Ohrr H: **The effect of methylenetetrahydrofolate reductase polymorphism C677T on cervical cancer in Korean women**. *Gynecol Oncol* 2004, **95**(3):557-563.
80. Tong SY, Kim MK, Lee JK, Lee JM, Choi SW, Friso S, Song ES, Lee KB, Lee JP: **Common polymorphisms in methylenetetrahydrofolate reductase gene are associated with risks of cervical intraepithelial neoplasia and cervical cancer in women with low serum folate and vitamin B12**. *Cancer Causes Control* 2011, **22**(1):63-72.
81. Zoodsma M, Nolte IM, Schipper M, Oosterom E, van der Steege G, de Vries EG, Te MG, van der Zee AG: **Methylenetetrahydrofolate reductase (MTHFR) and susceptibility for (pre)neoplastic cervical disease**. *Hum Genet* 2005, **116**(4):247-254.
82. Tong SY, Lee JM, Song ES, Lee KB, Kim MK, Yun YM, Lee JK, Son SK, Lee JP, Kim JH *et al*: **The effects of polymorphisms in methylenetetrahydrofolate reductase (MTHFR), methionine synthase (MTR), and methionine synthase reductase (MTRR) on the risk of cervical intraepithelial neoplasia and cervical cancer in Korean women**. *Cancer Causes Control* 2010, **21**(1):23-30.
83. Niwa Y, Matsuo K, Ito H, Hirose K, Tajima K, Nakanishi T, Nawa A, Kuzuya K, Tamakoshi A, Hamajima N: **Association of XRCC1 Arg399Gln and OGG1 Ser326Cys polymorphisms with the risk of cervical cancer in Japanese subjects**. *Gynecol Oncol* 2005, **99**(1):43-49.
84. De Ruyck K, Van Eijkeren M, Claes K, Morthier R, De Paepe A, Vral A, De Ridder L, Thierens H: **Radiation-induced damage to normal tissues after radiotherapy in patients treated for gynecologic tumors: association with single nucleotide polymorphisms in XRCC1, XRCC3, and OGG1 genes and in vitro chromosomal radiosensitivity in lymphocytes**. *Int J Radiat Oncol Biol Phys* 2005, **62**(4):1140-1149.
85. Lee JE, Lee SJ, Namkoong SE, Um SJ, Sull JW, Jee SH, You YK, Park JS: **Gene-gene and gene-environmental interactions of p53, p21, and IRF-1 polymorphisms in Korean women with cervix cancer**. *Int J Gynecol Cancer* 2004, **14**(1):118-125.
86. Bhattacharya P, Sengupta S: **Lack of evidence that proline homozygosity at codon 72 of p53 and rare arginine allele at codon 31 of p21, jointly mediate cervical cancer susceptibility among Indian women**. *Gynecol Oncol* 2005, **99**(1):176-182.
87. Roh JW, Kim BK, Lee CH, Kim J, Chung HH, Kim JW, Park NH, Song YS, Park SY, Kang SB: **P53 codon 72 and p21 codon 31 polymorphisms and susceptibility to cervical adenocarcinoma in Korean women**. *Oncol Res* 2010, **18**(9):453-459.
88. Roh J, Kim M, Kim J, Park N, Song Y, Kang S, Lee H: **Polymorphisms in codon 31 of p21 and cervical cancer susceptibility in Korean women**. *Cancer Lett* 2001, **165**(1):59-62.
89. Jiang P, Liu J, Li W, Zeng X, Tang J: **Role of p53 and p21 polymorphisms in the risk of cervical cancer among Chinese women**. *Acta Biochim Biophys Sin (Shanghai)* 2010, **42**(9):671-676.
90. Tian Q, Lu W, Chen H, Ye F, Xie X: **The nonsynonymous single-nucleotide polymorphisms in codon 31 of p21 gene and the susceptibility to cervical cancer in Chinese women**. *Int J Gynecol Cancer* 2009, **19**(6):1011-1014.
91. Wang N, Wang S, Zhang Q, Lu Y, Wei H, Li W, Zhang S, Yin D, Ou Y: **Association of p21 SNPs and risk of cervical cancer among Chinese women**. *Bmc Cancer* 2012, **12**:589.
92. Andersson S, Rylander E, Strand A, Sallstrom J, Wilander E: **The significance of p53 codon 72 polymorphism for the development of cervical adenocarcinomas**. *Br J Cancer* 2001, **85**(8):1153-1156.
93. Arbel-Alon S, Menczer J, Feldman N, Glezerman M, Yeremin L, Friedman E: **Codon 72 polymorphism of p53 in Israeli Jewish cervical cancer patients and healthy women**. *Int J Gynecol Cancer* 2002, **12**(6):741-744.
94. Bhattacharya P, Sengupta S: **Predisposition to HPV16/18-related cervical cancer because of proline homozygosity at codon 72 of p53 among Indian women is influenced by HLA-B*07 and homozygosity of HLA-DQB1*03**. *Tissue Antigens* 2007, **70**(4):283-293.
95. Cenci M, French D, Pisani T, Alderisio M, Lombardi AM, Marchese R, Colelli F, Vecchione A: **p53 polymorphism at codon 72 is not a risk factor for cervical carcinogenesis in central Italy**. *Anticancer Res* 2003, **23**(2B):1385-1387.
96. Dybikowska A, Dettlaff A, Konopa K, Podhajska A: **p53 codon 72 polymorphism in cervical cancer patients and healthy women from Poland**. *Acta Biochim Pol* 2000, **47**(4):1179-1182.
97. El KM, Ennaji MM, El KR, Mhand RA, Attaleb M, El MM: **p53 codon 72 polymorphism and risk of cervical carcinoma in Moroccan women**. *Med Oncol* 2010, **27**(3):861-866.
98. Ferreira DSI, Koifman RJ, Quinto SSC, Ferreira DANO, Koifman S: **TP53 genetic polymorphisms and environmental risk factors associated with cervical carcinogenesis in a cohort of Brazilian women with cervical lesions**. *J Toxicol Environ Health A* 2010, **73**(13-14):888-900.
99. Govan VA, Loubser S, Saleh D, Hoffman M, Williamson AL: **No relationship observed between human p53 codon-72 genotype and HPV-associated cervical cancer in a population group with a low arginine-72 allele frequency**. *Int J Immunogenet* 2007, **34**(3):213-217.
100. Gudleviciene Z, Didziapetriene J, Ramael M, Uleckiene S, Valuckas KP: **Human papillomavirus and p53 polymorphism in Lithuanian cervical cancer patients**. *Gynecol Oncol* 2006, **102**(3):530-533.
101. Gustafsson AC, Guo Z, Hu X, Ahmadian A, Brodin B, Nilsson A, Ponten J, Ponten F, Lundeberg J: **HPV-related cancer susceptibility and p53 codon 72 polymorphism**. *Acta Derm Venereol* 2001, **81**(2):125-129.
102. Eltahir HA, Elhassan AM, Ibrahim ME: **Contribution of retinoblastoma LOH and the p53 Arg/Pro polymorphism to cervical cancer**. *Mol Med Rep* 2012, **6**(3):473-476.
103. Humbey O, Aubin F, Cairey-Remonnay S, Riethmuller D, Pretet JL, Fest T, Seilles E, Mougin C: **TP53 polymorphism at exon 4 in caucasian women from eastern France: lack of correlation with HPV status and grade of cervical precancerous lesions**. *Eur J Obstet Gynecol Reprod Biol* 2002, **103**(1):60-64.
104. Jiang P, Liu J, Zeng X, Li W, Tang J: **Association of TP53 codon 72 polymorphism with cervical cancer risk in Chinese women**. *Cancer Genet Cytogenet* 2010, **197**(2):174-178.
105. Katiyar S, Thelma BK, Murthy NS, Hedau S, Jain N, Gopalkrishna V, Husain SA, Das BC: **Polymorphism of the p53 codon 72 Arg/Pro and the risk of HPV type 16/18-associated cervical and oral cancer in India**. *Mol Cell Biochem* 2003, **252**(1-2):117-124.
106. Kim JW, Roh JW, Park NH, Song YS, Kang SB, Lee HP: **Polymorphism of TP53 codon 72 and the risk of cervical cancer among Korean women**. *Am J Obstet Gynecol* 2001, **184**(2):55-58.
107. Klug SJ, Wilmotte R, Santos C, Almonte M, Herrero R, Guerrero I, Caceres E, Peixoto-Guimaraes D, Lenoir G, Hainaut P *et al*: **TP53 polymorphism, HPV infection, and risk of cervical cancer**. *Cancer Epidemiol Biomarkers Prev* 2001, **10**(9):1009-1012.
108. Koushik A, Ghosh A, Duarte-Franco E, Forest P, Voyer H, Matlashewski G, Coutlee F, Franco EL: **The p53 codon 72 polymorphism and risk of high-grade cervical intraepithelial neoplasia**. *Cancer Detect Prev* 2005, **29**(4):307-316.
109. Min-min H, Ming-rong X, Ze-yi C, Kai-xuan Y, Zhi-lin S: **Analysis of p53 codon 72 polymorphism and its association with human papillomavirus 16 and 18 E6 in Chinese cervical lesions**. *Int J Gynecol Cancer* 2006, **16**(6):2004-2008.
110. Mitra S, Misra C, Singh RK, Panda CK, Roychoudhury S: **Association of specific genotype and haplotype of p53 gene with cervical cancer in India**. *J Clin Pathol* 2005, **58**(1):26-31.
111. Niwa Y, Hamajima N, Atsuta Y, Yamamoto K, Tamakoshi A, Saito T, Hirose K, Nakanishi T, Nawa A, Kuzuya K *et al*: **Genetic polymorphisms of p73 G4C14-to-A4T14 at exon 2 and p53 Arg72Pro and the risk of cervical cancer in Japanese**. *Cancer Lett* 2004, **205**(1):55-60.
112. Pegoraro R, Moodley J, Naiker S, Lanning P, Rom L: **The p53 codon 72 polymorphism in black South African women and the risk of cervical cancer**. *BJOG* 2000, **107**(9):1164-1165.
113. Pegoraro RJ, Rom L, Lanning PA, Moodley M, Naiker S, Moodley J: **P53 codon 72 polymorphism and human papillomavirus type in relation to cervical cancer in South African women**. *Int J Gynecol Cancer* 2002, **12**(4):383-388.
114. Pillai MR, Sreevidya S, Pollock BH, Jayaprakash PG, Herman B: **Polymorphism at codon 72 of p53, human papillomavirus, and cervical cancer in South India**. *J Cancer Res Clin Oncol* 2002, **128**(11):627-631.
115. Roh JW, Kim BK, Lee CH, Kim J, Chung HH, Kim JW, Park NH, Song YS, Park SY, Kang SB: **P53 codon 72 and p21 codon 31 polymorphisms and susceptibility to cervical adenocarcinoma in Korean women**. *Oncol Res* 2010, **18**(9):453-459.
116. Santos AM, Sousa H, Pinto D, Portela C, Pereira D, Catarino R, Duarte I, Lopes C, Medeiros R: **Linking TP53 codon 72 and P21 nt590 genotypes to the development of cervical and ovarian cancer**. *Eur J Cancer* 2006, **42**(7):958-963.
117. Saranath D, Khan Z, Tandle AT, Dedhia P, Sharma B, Contractor R, Shrivastava S, Dinshaw K: **HPV16/18 prevalence in cervical lesions/cancers and p53 genotypes in cervical cancer patients from India**. *Gynecol Oncol* 2002, **86**(2):157-162.
118. Settheetham-Ishida W, Singto Y, Yuenyao P, Tassaneeyakul W, Kanjanavirojkul N, Ishida T: **Contribution of epigenetic risk factors but not p53 codon 72 polymorphism to the development of cervical cancer in Northeastern Thailand**. *Cancer Lett* 2004, **210**(2):205-211.
119. Settheetham-Ishida W, Kanjanavirojkul N, Kularbkaew C, Ishida T: **Human papillomavirus genotypes and the p53 codon 72 polymorphism in cervical cancer of Northeastern Thailand**. *Microbiol Immunol* 2005, **49**(5):417-421.
120. Settheetham-Ishida W, Yuenyao P, Tassaneeyakul W, Kanjanavirojkul N, Thawmor A, Kularbkaew C, Hahvajanawong C, Settheetham D, Wattanathorn J, Kashima T *et al*: **Selected risk factors, human papillomavirus infection and the p53 codon 72 polymorphism in patients with squamous intraepithelial lesions in northeastern Thailand**. *Asian Pac J Cancer Prev* 2006, **7**(1):113-118.
121. Tanara G, Falugi C, Cesario A, Margaritora S, Russo P, Cosimi A: **TP53 codon 72 polymorphism does not affect risk of cervical cancer in patients from The Gambia**. *Int J Biol Markers* 2003, **18**(4):280-283.
122. Ye F, Zhang J, Cheng Q, Shen J, Chen H: **p53 Codon 72 polymorphism is associated with occurrence of cervical carcinoma in the Chinese population**. *Cancer Lett* 2010, **287**(1):117-121.
123. Zheng XZ, Yang AQ, Pan XL, Zheng LL, Wang XL, Zhou QY, Li XM, Yan LH, Zhang B, Li HA *et al*: **Ethnicity determines association of p53Arg72Pro alleles with cervical cancer in China**. *Eur J Cancer Prev* 2008, **17**(5):460-466.
124. Zhou X, Han S, Wang S, Chen X, Dong J, Shi X, Xia Y, Wang X, Hu Z, Shen H: **Polymorphisms in HPV E6/E7 protein interacted genes and risk of cervical cancer in Chinese women: a case-control analysis**. *Gynecol Oncol* 2009, **114**(2):327-331.
125. Craveiro R, Bravo I, Catarino R, Teixeira AL, Sousa H, Pereira D, Pereira H, Medeiros R: **The role of p73 G4C14-to-A4T14 polymorphism in the susceptibility to cervical cancer**. *Dna Cell Biol* 2012, **31**(2):224-229.
126. Zhou X, Chen X, Hu L, Han S, Qiang F, Wu Y, Pan L, Shen H, Li Y, Hu Z: **Polymorphisms involved in the miR-218-LAMB3 pathway and susceptibility of cervical cancer, a case-control study in Chinese women**. *Gynecol Oncol* 2010, **117**(2):287-290.
127. Shi TY, Chen XJ, Zhu ML, Wang MY, He J, Yu KD, Shao ZM, Sun MH, Zhou XY, Cheng X *et al*: **A pri-miR-218 variant and risk of cervical carcinoma in Chinese women**. *Bmc Cancer* 2013, **13**:19.
128. Lai ZZ, Ni-Zhang, Pan XL, Song L: **Toll-like receptor 9 (TLR9) gene polymorphisms associated with increased susceptibility of human papillomavirus-16 infection in patients with cervical cancer**. *J Int Med Res* 2013, **41**(4):1027-1036.
129. Pandey S, Mittal B, Srivastava M, Singh S, Srivastava K, Lal P, Mittal RD: **Evaluation of Toll-like receptors 3 (c.1377C/T) and 9 (G2848A) gene polymorphisms in cervical cancer susceptibility**. *Mol Biol Rep* 2011, **38**(7):4715-4721.
130. Roszak A, Lianeri M, Sowinska A, Jagodzinski PP: **Involvement of Toll-like Receptor 9 polymorphism in cervical cancer development**. *Mol Biol Rep* 2012, **39**(8):8425-8430.
131. Chen X, Wang S, Liu L, Chen Z, Qiang F, Kan Y, Shen Y, Wu J, Shen H, Hu Z: **A genetic variant in the promoter region of Toll-like receptor 9 and cervical cancer susceptibility**. *Dna Cell Biol* 2012, **31**(5):766-771.
132. Duarte I, Santos A, Sousa H, Catarino R, Pinto D, Matos A, Pereira D, Moutinho J, Canedo P, Machado JC *et al*: **G-308A TNF-alpha polymorphism is associated with an increased risk of invasive cervical cancer**. *Biochem Biophys Res Commun* 2005, **334**(2):588-592.
133. Wang N, Yin D, Zhang S, Wei H, Wang S, Zhang Y, Lu Y, Dai S, Li W, Zhang Q *et al*: **TNF-alpha rs1800629 polymorphism is not associated with HPV infection or cervical cancer in the Chinese population**. *PLoS One* 2012, **7**(9):e45246.
134. Kohaar I, Thakur N, Salhan S, Batra S, Singh V, Sharma A, Sodhani P, Das BC, Sarkar DP, Bharadwaj M: **TNFalpha-308G/A polymorphism as a risk factor for HPV associated cervical cancer in Indian population**. *Cell Oncol* 2007, **29**(3):249-256.
135. Govan VA, Constant D, Hoffman M, Williamson AL: **The allelic distribution of -308 Tumor Necrosis Factor-alpha gene polymorphism in South African women with cervical cancer and control women**. *Bmc Cancer* 2006, **6**:24.
136. Jang WH, Yang YI, Yea SS, Lee YJ, Chun JH, Kim HI, Kim MS, Paik KH: **The -238 tumor necrosis factor-alpha promoter polymorphism is associated with decreased susceptibility to cancers**. *Cancer Lett* 2001, **166**(1):41-46.
137. Stanczuk GA, Sibanda EN, Tswana SA, Bergstrom S: **Polymorphism at the -308-promoter position of the tumor necrosis factor-alpha (TNF-alpha) gene and cervical cancer**. *Int J Gynecol Cancer* 2003, **13**(2):148-153.
138. Deshpande A, Nolan JP, White PS, Valdez YE, Hunt WC, Peyton CL, Wheeler CM: **TNF-alpha promoter polymorphisms and susceptibility to human papillomavirus 16-associated cervical cancer**. *J Infect Dis* 2005, **191**(6):969-976.
139. Gostout BS, Poland GA, Calhoun ES, Sohni YR, Giuntoli RN, McGovern RM, Sloan JA, Cha SS, Persing DH: **TAP1, TAP2, and HLA-DR2 alleles are predictors of cervical cancer risk**. *Gynecol Oncol* 2003, **88**(3):326-332.
140. Wang SS, Purdue MP, Cerhan JR, Zheng T, Menashe I, Armstrong BK, Lan Q, Hartge P, Kricker A, Zhang Y *et al*: **Common gene variants in the tumor necrosis factor (TNF) and TNF receptor superfamilies and NF-kB transcription factors and non-Hodgkin lymphoma risk**. *PLoS One* 2009, **4**(4):e5360.
141. Kim YH, Kim MA, Park IA, Park WY, Kim JW, Kim SC, Park NH, Song YS, Kang SB: **VEGF polymorphisms in early cervical cancer susceptibility, angiogenesis, and survival**. *Gynecol Oncol* 2010, **119**(2):232-236.
142. Konac E, Onen HI, Metindir J, Alp E, Biri AA, Ekmekci A: **Lack of association between -460 C/T and 936 C/T of the vascular endothelial growth factor and angiopoietin-2 exon 4 G/A polymorphisms and ovarian, cervical, and endometrial cancers**. *Dna Cell Biol* 2007, **26**(7):453-463.
143. Barbisan G, Perez LO, Difranza L, Fernandez CJ, Ciancio NE, Golijow CD: **XRCC1 Arg399Gln polymorphism and risk for cervical cancer development in Argentine women**. *Eur J Gynaecol Oncol* 2011, **32**(3):274-279.
144. Cheng XD, Lu WG, Ye F, Wan XY, Xie X: **The association of XRCC1 gene single nucleotide polymorphisms with response to neoadjuvant chemotherapy in locally advanced cervical carcinoma**. *J Exp Clin Cancer Res* 2009, **28**:91.
145. Farkasova T, Gurska S, Witkovsky V, Gabelova A: **Significance of amino acid substitution variants of DNA repair genes in radiosusceptibility of cervical cancer patients; a pilot study**. *Neoplasma* 2008, **55**(4):330-337.
146. Huang J, Ye F, Chen H, Lu W, Xie X: **The nonsynonymous single nucleotide polymorphisms of DNA repair gene XRCC1 and susceptibility to the development of cervical carcinoma and high-risk human papillomavirus infection**. *Int J Gynecol Cancer* 2007, **17**(3):668-675.
147. Roszak A, Lianeri M, Jagodzinski PP: **Involvement of the XRCC1 Arg399Gln gene polymorphism in the development of cervical carcinoma**. *Int J Biol Markers* 2011, **26**(4):216-220.
148. Settheetham-Ishida W, Yuenyao P, Natphopsuk S, Settheetham D, Ishida T: **Genetic risk of DNA repair gene polymorphisms (XRCC1 and XRCC3) for high risk human papillomavirus negative cervical cancer in Northeast Thailand**. *Asian Pac J Cancer Prev* 2011, **12**(4):963-966.
149. Wu MT, Chen SY, Wu TN, Hwang HY, Ho CK, Lee LH, Wu SC: **No association between polymorphisms of the DNA repair geneXRCC1 and cervical neoplasm risk**. *Environ Health Prev Med* 2003, **8**(3):100-103.
150. Wu MT, Liu CL, Ho CK, Wu TN: **Genetic polymorphism of p53 and XRCC1 in cervical intraepithelial neoplasm in Taiwanese women**. *J Formos Med Assoc* 2004, **103**(5):337-343.
151. Kim K, Kang SB, Chung HH, Kim JW, Park NH, Song YS: **XRCC1 Arginine194Tryptophan and GGH-401Cytosine/Thymine polymorphisms are associated with response to platinum-based neoadjuvant chemotherapy in cervical cancer**. *Gynecol Oncol* 2008, **111**(3):509-515.
152. He X, Ye F, Zhang J, Cheng Q, Shen J, Chen H: **Susceptibility of XRCC3, XPD, and XPG genetic variants to cervical carcinoma**. *Pathobiology* 2008, **75**(6):356-363.
