# Supplementary material for: Association of 42 SNPs with genetic risk for cervical cancer: an extensive meta-analysis
Source: BMC Med Genet. 2015 Apr 15;16:25. doi: 10.1186/s12881-015-0168-z (PMC4436168; doi:10.1186/s12881-015-0168-z)

Forest plots and Funnel plots

1. CYP1A1 rs1048943 G/A allele model


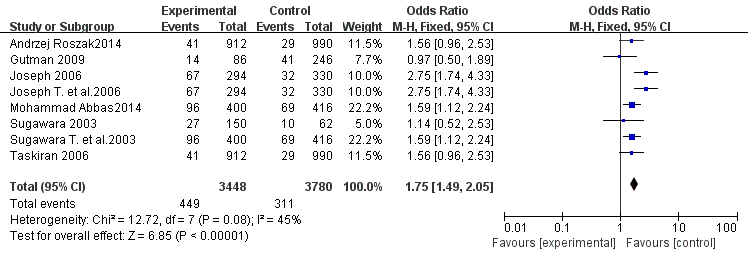

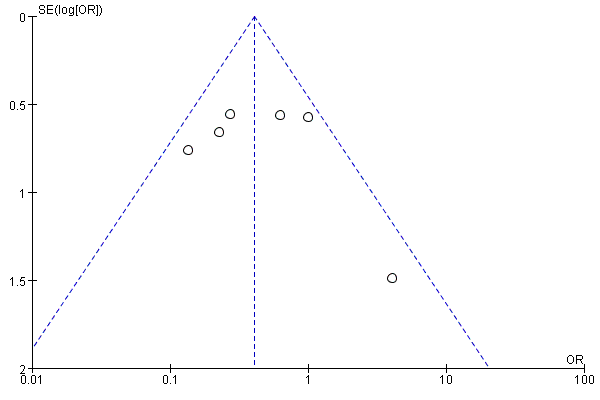


1. CYP1A1 rs1048943 GG+GA/AA dominant genetic model


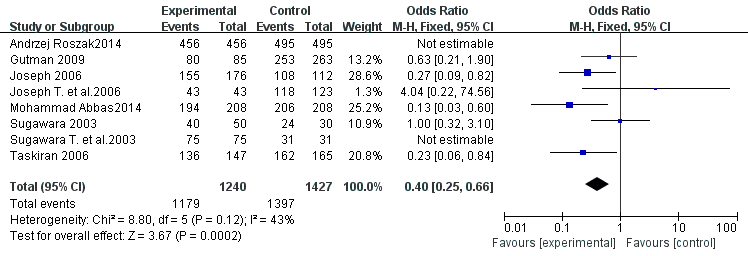

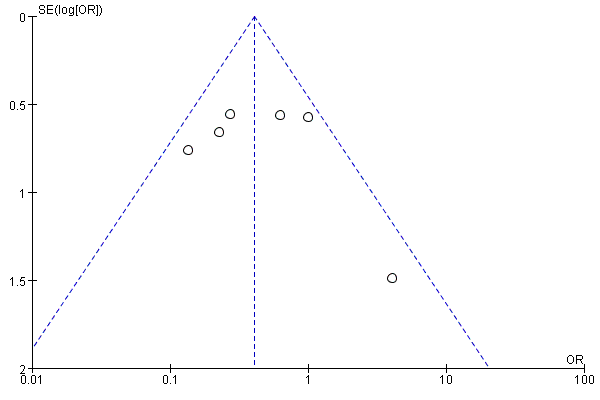


1. CYP1A1 rs1048943 GG/GA+AA recessive genetic model


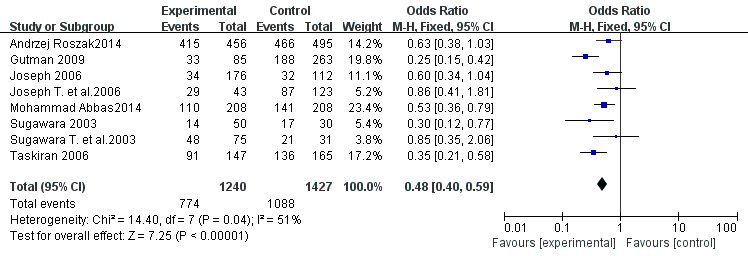

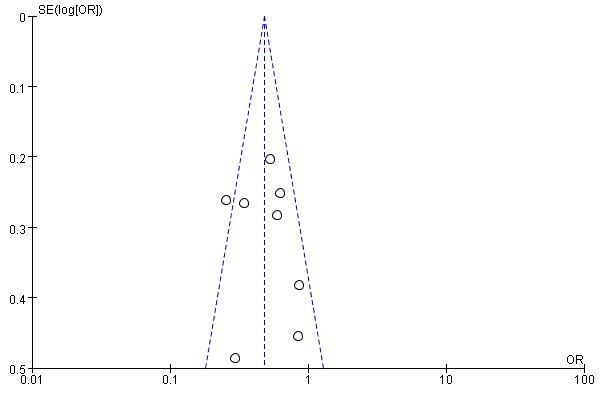


1. CYP1A1 rs4646903 CC/CA+AA recessive genetic model


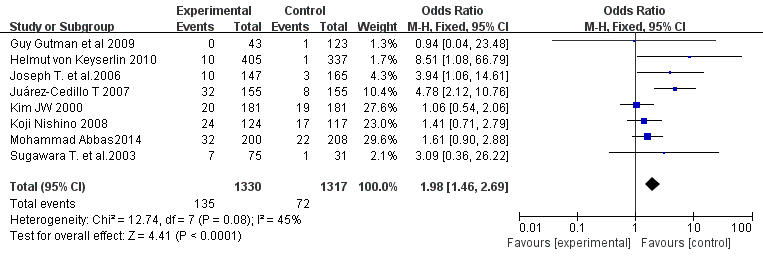

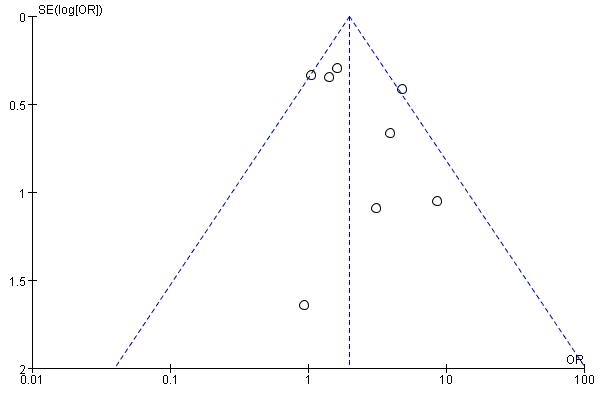


1. IL-1β rs16944 CC/CT+TT recessive genetic model


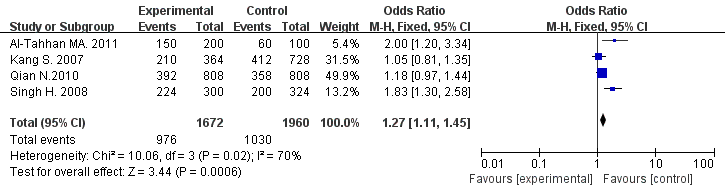

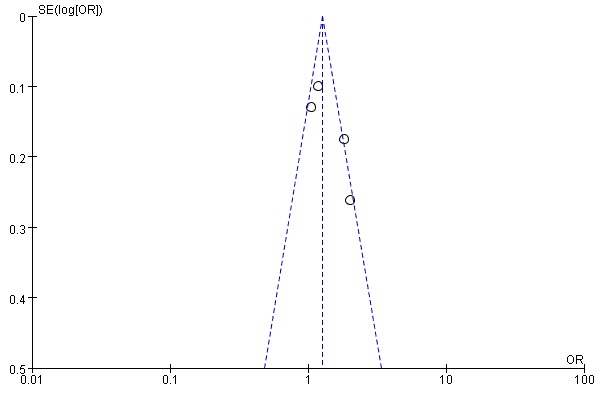

Supplement: Additional file 3: — Forest plots and Funnel plots. [file 12881_2015_168_MOESM3_ESM.docx]
