# Supplementary material for: Association of 42 SNPs with genetic risk for cervical cancer: an extensive meta-analysis
Source: BMC Med Genet. 2015 Apr 15;16:25. doi: 10.1186/s12881-015-0168-z (PMC4436168; doi:10.1186/s12881-015-0168-z)

Sensitivity analysis

1. CTLA-318 rs5742909 in dominant genetic model (All studies included)


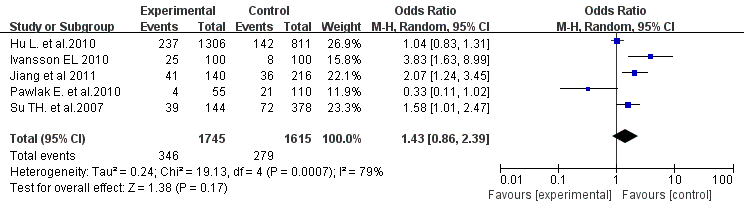


CTLA-318 rs5742909 in dominant genetic model (Pawlak E 2010removed)


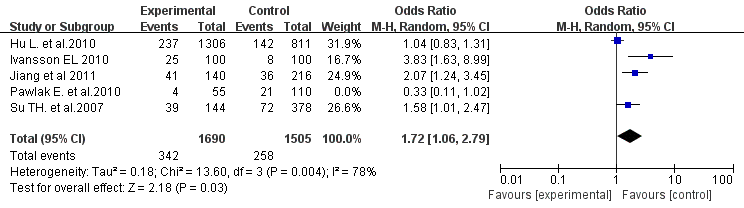


1. XRCC1 codon 194 in dominant genetic model (All studies included)


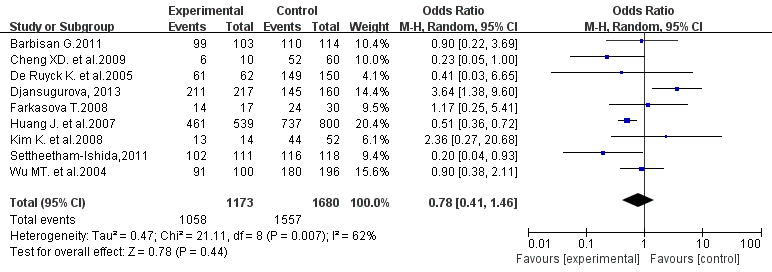


XRCC1 codon 194 in dominant genetic model (Djansugurova 2005 removed)


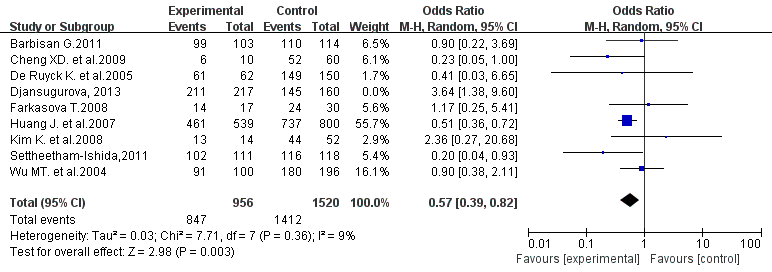


1. IFN-r rs62559044 4 in allele model (All studies included)


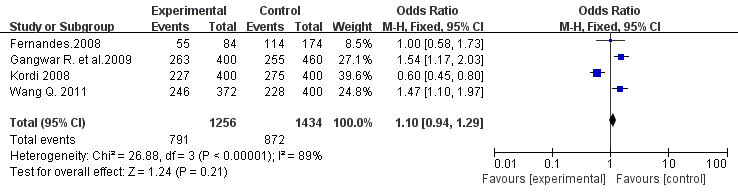


IFN-r rs62559044 4 in allele model (Kordi 2008)


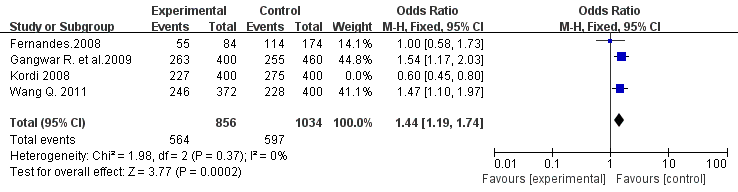

Supplement: Additional file 4: — Sensitivity analysis. [file 12881_2015_168_MOESM4_ESM.docx]
